# A review of valve surgery for rheumatic heart disease in Australia

**DOI:** 10.1186/1471-2261-14-134

**Published:** 2014-10-02

**Authors:** Elizabeth Anne Russell, Lavinia Tran, Robert A Baker, Jayme S Bennetts, Alex Brown, Christopher Michael Reid, Robert Tam, Warren Frederick Walsh, Graeme Paul Maguire

**Affiliations:** Baker IDI Central Australia, PO Box 1294, Alice Springs, NT 0811 Australia; School of Epidemiology and Preventive Medicine, Monash University, Melbourne, Victoria Australia; Department of Cardiac and Thoracic Surgery, Flinders Medical Centre, Adelaide, SA Australia; Department of Surgery, School of Medicine, Flinders University, Adelaide, SA Australia; Wardliparingga Aboriginal Research Unit, South Australia Health and Medical Research Institute, Adelaide, SA Australia; School of Population Health, University of South Australia, Adelaide, SA Australia; Department of Cardiothoracic Surgery, The Townsville Hospital, Queensland, Australia; Cardiology Department, Prince of Wales Hospital, Randwick, NSW Australia; School of Medicine, James Cook University, Cairns, Queensland Australia

**Keywords:** Rheumatic heart disease, Rheumatic valve surgery, Indigenous health, Valve choice

## Abstract

**Background:**

Globally, rheumatic heart disease (RHD) remains an important cause of heart disease. In Australia it particularly affects older non-Indigenous Australians and Aboriginal Australians and/or Torres Strait Islander peoples. Factors associated with the choice of treatment for advanced RHD remain variable and poorly understood.

**Methods:**

The Australian and New Zealand Society of Cardiac and Thoracic Surgeons Cardiac Surgery Database was analysed. Demographics, co-morbidities, pre-operative status and valve(s) affected were collated and associations with management assessed.

**Results:**

Surgical management of 1384 RHD and 15843 non-RHD valve procedures was analysed. RHD patients were younger, more likely to be female and Indigenous Australian, to have atrial fibrillation (AF) and previous percutaneous balloon valvuloplasty (PBV). Surgery was performed on one valve in 64.5%, two valves in 30.0% and three valves in 5.5%. Factors associated with receipt of mechanical valves in RHD were AF (OR 2.69) and previous PBV (OR 1.98) and valve surgery (OR 3.12). Predictors of valve repair included being Indigenous (OR 3.84) and having fewer valves requiring surgery (OR 0.10). Overall there was a significant increase in the use of mitral bioprosthetic valves over time.

**Conclusions:**

RHD valve surgery is more common in young, female and Indigenous patients. The use of bioprosthetic valves in RHD is increasing. Given many patients are female and younger, the choice of valve surgery and need for anticoagulation has implications for future management of RHD and related morbidity, pregnancy and lifestyle plans.

## Background

Rheumatic heart disease (RHD) is a condition of global health importance. It is estimated 15.6 - 19.6 million people are living with RHD, with almost 80% of those residing in low and middle-income countries [1, 2]. Whilst RHD is now rare in high income countries [3], it remains an important cause of preventable heart disease in some Indigenous populations in these countries. This is likely to be explained by a combination of educational, economic and environmental disadvantage and reduced access to primary and specialist health care [4]. In 2010 the prevalence of RHD amongst Australia’s Aboriginal and Torres Strait Islander Indigenous peoples was 6.45 per 1000 or 26 times that of non-Indigenous Australians [5]. A recent echocardiographic screening study of Indigenous Australian children aged 5–14 years, found a prevalence of definite RHD [6] of 8.6 per 1000 (95% CI 6.0-12.0) with none detected in a comparably aged non-Indigenous cohort [7].

In some populations at risk of RHD, such as Aboriginal Australians and Torres Strait Islanders, outcomes following cardiac surgery can be inferior [8, 9] despite being of younger age at time of surgery [8]. This is likely to be related to factors including comorbidities [4, 8, 9], barriers to primary and specialist health care and the ability to achieve safe anticoagulation during long-term follow-up [10].

The most common heart valves affected by RHD and non-RHD causes are the mitral and aortic valves, less commonly the tricuspid and rarely the pulmonary valve. Rheumatic valve disease most commonly leads to regurgitation [6, 11] and less commonly to valve stenosis or mixed regurgitation and stenosis [12]. Although the majority of rheumatic valve disease cases are only mildly affected, [1] a minority progress to more severe disease requiring valve surgery [13].

The options for surgical management of rheumatic valve disease are valve repair or replacement with either a bioprosthetic or mechanical prosthesis. In patients with mitral stenosis an additional option is non-surgical percutaneous mitral balloon valvuloplasty [12, 14]. There are limited data available about factors which might affect the choice of surgery in patients with rheumatic valve disease. This decision is likely to be influenced by patient geography, medication access and use, timing and venue of referral, gender and access to ongoing care and follow-up. There have been no Australian multi-centre studies of rheumatic valve surgery published with most published data pertaining to small single centre series.

The aim of this study was thus to examine the Australian patient population having valve surgery for RHD and review the pre-operative factors associated with the choice of surgical management of RHD in Australia.

## Methods

### The database

The Australia and New Zealand Society of Cardiac and Thoracic Surgeons (ANZSCTS) National Cardiac Surgery Database is an Australia-wide database for the collection and analysis of cardiac surgical procedures, established to enable benchmarking and comparison with international standards [15]. The database definition set was developed by the ANZSCTS for all participating cardiac surgery units. There is an opt-out Patient Information Sheet which has the approval of each site’s Human Research Ethics Committee. At present 19 of 25 Australian public hospital cardiac surgical units enter data relating to cardiac surgical procedures that identify whether patients are Aboriginal Australians and/or Torres Strait Islanders.

The database collects patient demographics, co-morbidities, pre-operative status, previous interventions, haemodynamic data, surgery type and surgical and post-operative outcome data. Only de-identified data is abstracted and utilised for analysis.

### Analysis

The aim of the analysis was to describe patients having valve surgery for rheumatic valve disease, to compare Aboriginal and Torres Strait Islander RHD patients with non-Indigenous Australians and to describe and identify factors associated with treatment choice. Demographic data included age, gender, Indigenous status, concomitant coronary artery bypass grafting (CABG) and rurality by Remoteness Area (RA) category as defined by the Australian Statistical Geography Standard [16]. Co-morbidities assessed included chronic kidney disease (defined as pre-operative estimated glomerular filtration rate (eGFR) less than 60 mL/min/1.73 m^2^ based on the Modification of Diet in Renal Disease (MDRD) equation and stratified to stages 3 (30 - 59 mL/min/1.73 m^2^), 4 (15 - 29 mL/min/1.73 m^2^), and 5 (<15 mL/min/1.73 m^2^) [17], elevated (200 μmol/L or more) pre-surgery serum creatinine, a pre-existing clinician diagnosis of diabetes mellitus and hypertension and smoking status.

The pre-operative status relating to underlying heart disease included symptomatic status based on the New York Heart Association (NYHA) classes I to IV [18], pre-operative atrial fibrillation, echocardiographic assessment of left ventricular ejection fraction (LVEF) (stratified to more than 45%, 30% - 45% and less than 30%), previous valve surgery and percutaneous balloon valvuloplasty (PBV). Valvular lesions were analysed according to the valve(s) affected, the valvular lesion (regurgitation, stenosis or mixed), the number of valves affected, and the year of surgery. Valve-related surgical procedure data included valve repair or replacement and in the case of replacement, whether this was a mechanical or bioprosthetic valve.

### Statistical analysis

Data were analysed using IBM SPSS Statistics 20 (IBM, New York, USA) and Stata 13 (StataCorp LP, Texas, USA). Descriptive data were summarised using standard univariate techniques and reported as percentages with 95% confidence intervals (95% CI), means with standard deviation (SD) or medians with interquartile range (IQR) depending on the data format and distribution. Comparisons between groups were undertaken using χ^2^ for categorical data and Student’s t-Test or Mann–Whitney U test for continuous Normally distributed or non-Normally distributed data respectively. A p valve less than 0.05 was taken to indicate statistical significance and all tests were two-sided.

Logistic regression models were developed to identify independent factors associated with the type of valve surgical procedure utilised. These were developed using a backwards stepwise approach including in the first model all factors associated with a particular management choice using bivariate analysis with a p value <0.1. Factors with a p value > =0.05 were progressively removed from the models starting with the variable with an odds ratio (OR) closest to 1. Interactions between predictive factors were explored and final models were limited to predictive factors with significant coefficients (p < 0.05).

Approval for this project was granted by the Monash University Human Research Ethics Committee (CF13/2737 – 2013001472).

## Results

**Figure 1:**
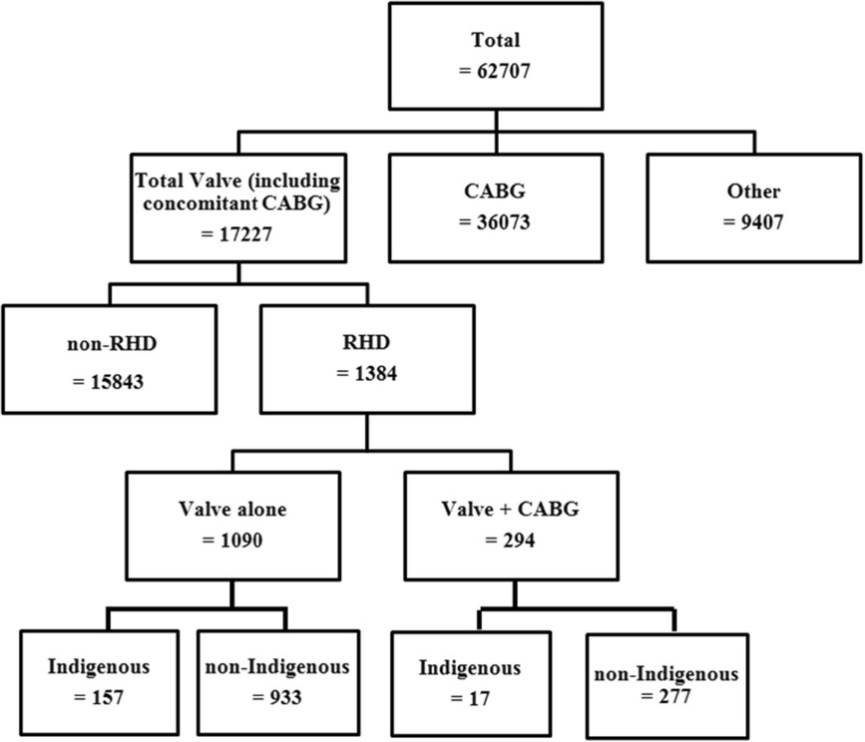
**Cardiac surgical procedures collected in the ANZCTS Database between 1 August 2001 and 31 December 2012.**

Data in relation to 62 707 cardiac surgical procedures performed between 1 August 2001 and 31 December 2012 were analysed. A breakdown of those procedures is summarized in Figure 1.

**Figure 2:**
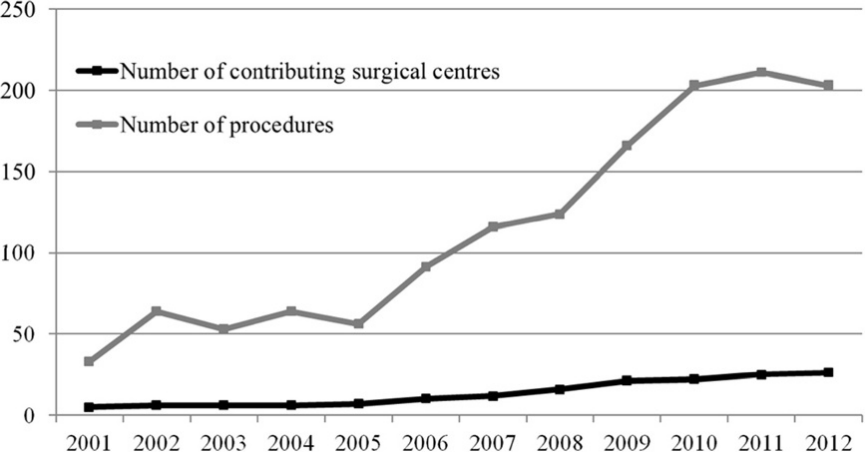
**Number of contributing surgical centres and RHD valve procedures over time.**

A subset of 17 227 surgical valve procedures with or without coronary artery bypass grafting (CABG) was included for analysis. Contributing surgical centres have increased from five in 2001 with 33 RHD valve surgeries to 26 in 2012 and 203 RHD valve surgeries (Figure 2).

Descriptive characteristics of these valve surgery patients are outlined in Table 1. RHD valve surgery patients were, compared with non-RHD valve surgery patients, younger, more likely to be female and Aboriginal and/or Torres Strait Islander and were less likely to have concomitant CABG, severely impaired left ventricular systolic function (LVEF <30%) or associated diabetes or hypertension. RHD patients were also more likely to have associated atrial fibrillation (AF), be current smokers or have a past history of valve surgery and/or PBV.

**Table 1:** **Descriptive characteristics of valve surgery patients stratified by causation**

	All	RHD-related	Non-RHD	P value
	N = 17227	N = 1384	N = 15843	
Age (years)	71.3	59.7	71.9	<0.001
(Median (IQR))	(61.2 – 78.3)	(50.9 – 71.4)	(62.3 – 78.6)	
Sex (% female)	37.3	64.5	35.0	<0.001
(95% CI)	(36.6 – 38.1)	(61.9 – 67.0)	(34.2 – 35.7)	
Indigenous status	1.9	12.6	1.0	<0.001
(% Aboriginal and Torres Strait Islander people) (95% CI)	(1.7 – 2.1)	(10.9 – 14.4)	(0.8 – 1.2)	
Concomitant CABG	39.1	21.2	40.7	<0.001
(%, 95% CI)	(38.4 – 39.8)	(19.1 – 23.5)	(39.9 – 41.4)	
Pre-operative comorbidities				
Diabetes	23.2	20.3	23.4	0.009
(%, 95% CI)	(22.5 – 23.8)	(18.2 – 22.5)	(22.8 – 24.1)	
Elevated Creatinine	3.4	2.8	3.5	0.436
(% Cr > =200 μmol/L, 95% CI)	(3.1 – 3.7)	(2.0 – 03.8)	(3.2 – 03.8)	
Chronic kidney disease	36.7	31.2	37.2	0.814
(% eGFR < 60 mL/min/1.73 m^2^) (95% CI)	(36.0 – 37.5)	(28.8 – 33.7)	(36.5 – 38.0)	
Hypertension	67.0	53.0	68.2	<0.001
(%, 95% CI)	(66.3 – 67.7)	(50.3 – 55.7)	(67.5 – 68.9)	
Previous smoking	53.1	52.7	53.1	0.955
(%, 95% CI)	(52.3 -53.8)	(50.0 - 55.3)	(52.3 - 53.9)	
Current smoking	16.0	25.1	15.2	<0.001
(%, 95% CI)	(15.2 - 16.7)	(22.0 - 28.4)	(14.5 - 16.0)	
Pre-operative status				
NYHA classes III & IV	43.7	53.7	42.8	0.351
(%, 95% CI)	(42.9 – 44.4)	(51.0 – 56.4)	(42.0 – 43.6)	
Atrial fibrillation	19.3	40.5	17.4	<0.001
(%, 95% CI)	(18.7 – 19.9)	(37.9 – 43.2)	(16.8 – 18.0)	
LVEF >45%	81.2	84.6	80.9	0.001
(%, 95% CI)	(80.6 – 81.8)	(82.6 – 86.5)	(80.3 – 81.5)	
LVEF 45 – 60%	12.1	10.9	12.2	0.154
(%, 95% CI)	(11.6 – 12.6)	(9.3 – 12.7)	(11.7 – 12.7)	
LVEF <30%	4.3	2.2	4.5	<0.001
(%, 95% CI)	(4.0 – 4.6)	(1.5 – 3.2)	(4.2 – 4.8)	
Previous procedures				
Valve surgery	6.4	13.5	5.8	<0.001
(%, 95% CI)	(6.1 – 6.8)	(11.8 – 15.4)	(5.4 – 6.2)	
PBV	4.9	20.7	3.3	<0.001
(%, 95% CI)	(4.3 – 5.6)	(16.7 – 25.2)	(2.8 – 4.0)	

In multivariate modeling, patients undergoing RHD-related surgery were younger (OR 0.99/additional year, 95% CI 0.97 – 1.00), more likely to be female (OR 4.15, 95% CI 3.00 – 5.75), Aboriginal and/or Torres Strait Islander (OR 5.10, 95% CI 2.67 – 9.80), have associated AF (OR 3.85, 95% CI 2.72 – 5.44), a history of PBV (OR 5.71, 95% CI 3.37 – 9.71) or prior valve surgery (OR 1.81, 95% CI 1.26 – 2.60), and were less likely to have hypertension (OR 0.67, 95% CI 0.46 – 1.00) or severe left ventricular dysfunction (OR 0.17, 95% CI 0.05 – 0.58). Details regarding RHD valve surgery patients, stratified by Indigenous status, are outlined in Table 2. In bivariate analyses Aboriginal Australian and/or Torres Strait Islander RHD valve surgery patients were, compared with non- Indigenous Australian patients, younger and less likely to have concomitant CABG, associated chronic kidney disease, hypertension or AF. They were also more likely to be previous or current smokers and to be living in remote Australia. In multivariate logistic regression modeling, Indigenous Australian patients were younger (OR 0.89/additional year, 95% CI 0.87 – 0.91), current smokers (OR 2.52, 95% CI 1.40 – 4.51), residents of remote Australia (OR 15.39, 95% CI 7.81 – 30.30) and, in contrast to bivariate analysis, were more likely to have associated hypertension (OR 1.87, 95% CI 1.04 – 3.39), chronic kidney disease (OR 2.22, 95% CI 1.07 – 4.59) and AF (OR 2.09, 95% CI 1.17 – 3.71) once age was controlled for. There were no significant independent interactions between these factors.

**Table 2:** **Descriptive characteristics of RHD valve surgery patients stratified by Indigenous status**

	Aboriginal and/or Torres Strait Islander	Non-Indigenous Australian	P value
	N = 174	N = 1210	
Age (years)	37.4	65.1	<0.001
(Median (IQR))	(26.9 – 49.1)	(55.5 – 72.8)	
Sex (% female)	67.2	64.0	0.411
(95% CI)	(59.7 – 74.2)	(61.3 – 66.8)	
Concomitant CABG	9.8	22.9	<0.001
(%, 95% CI)	(5.8 – 15.2)	(20.6 – 25.4)	
Area of residence			
Remote and very remote	54.1	1.6	<0.001
(% RA category 3 & 4, 95% CI)	(46.3 – 61.7)	(1.0 – 2.4)	
Inner and outer regional	39.5	33.3	0.108
(% RA category 1 & 2, 95% CI)	(32.2 – 47.3)	(30.7 – 36.1)	
Major city	6.4	65.1	<0.001
(%, 95% CI)	(3.2 – 11.2)	(62.3 – 67.8)	
Pre-surgery comorbidities			
Diabetes	24.3	19.8	0.167
(%, 95% CI)	(18.1 – 31.4)	(17.5 – 22.1)	
Elevated Creatinine	2.9	2.7	0.912
(% Cr > =200 μmol/L) (95% CI)	(0.9 – 6.6)	(1.9 – 3.8)	
Chronic kidney disease (% eGFR < 60 mL/min/1.73 m^2^)	14.4	33.5	<0.001
(95% CI)	(9.5 – 20.5)	(30.8 – 36.2)	
Hypertension	37.0	55.3	<0.001
(%, 95% CI)	(29.8 – 44.7)	(52.4 – 58.1)	
Previous smoking	64.2	51.0	<0.001
(%, 95% CI)	(56.5 – 71.3)	(48.2 – 53.9)	
Current smoking	55.4	19.7	<0.001
(%, 95% CI)	(45.7 – 64.8)	(16.7 – 23.1)	
Pre-operative status			
NYHA classes III & IV	47.1	53.1	0.138
(%, 95% CI)	(39.5 – 54.8)	(50.3 – 56.0)	
Atrial fibrillation	33.3	41.6	0.039
(%, 95% CI)	(26.4 – 40.9)	(38.8 – 44.4)	
LVEF >45%	83.9	84.7	0.784
(%, 95% CI)	(77.6 – 89.0)	(82.6 – 86.7)	
LVEF 30 – 45%	11.5	10.8	0.792
(%, 95% CI)	(7.2 – 17.2)	(9.1 – 12.7)	
LVEF <30%	3.4	2.1	0.249
(%, 95% CI)	(1.3 – 7.4)	(1.3 – 3.1)	
Previous procedures			
Valve surgery	16.1	13.1	0.287
(%, 95% CI)	(11.0 – 22.4)	(11.3 – 15.2)	
PBV	29.5	19.5	0.124
(%, 95% CI)	(16.8 – 45.2)	(15.3 – 24.3)	

Of patients having RHD valve surgery, 64.5% (95% CI 61.91 – 67.02) required surgery on one valve only, 30.0% (95% CI 27.60 – 32.50) on two valves and 5.5% (95% CI 4.35 – 6.83) on three valves. The details of the valves involved and the associations between different valvular involvement is outlined in Table 3. RHD pulmonary valve only surgery accounted for only 0.3% (95% CI: 0.08 – 0.60) of procedures and combined RHD aortic, mitral and pulmonary valve surgery, 0.1% (95% CI: 0.02 – 0.52).

**Table 3:** **Association between different RHD-related valve disease requiring surgical management**

% (95% CI)		
**1 valve**		
Mitral valve only	40.3	(37.7 – 42.9)
Aortic valve only	22.9	(20.7 – 25.2)
Tricuspid valve only	1.2	(0.7 – 2.0)
**2 valves**		
Mitral and aortic valves	20.6	(18.5 – 22.8)
Mitral and tricuspid valves	8.5	(7.1 – 10.1)
Aortic and tricuspid valves	0.7	(0.3 – 1.3)
**3 valves**		
Mitral, aortic and tricuspid valves	5.4	(4.2 – 6.7)

The choice of surgical valve procedure for aortic, mitral and tricuspid valve disease overall and stratified by Indigenous status is outlined in Table 4. In bivariate analyses, Indigenous patients were less likely to have mechanical mitral valve replacement, more likely to have mitral valve repair and less likely to have bioprosthetic aortic valve replacement.

**Table 4:** **Surgical management of RHD valve disease stratified by Indigenous status** (**There were only five pulmonary procedures performed**; **all on non**-**Indigenous patients**)

% (95% CI)	Total RHD	Aboriginal and/or Torres Strait Islander	Non-Indigenous Australian	P value
	N = 1384	N = 174	N = 1210	
Mitral		N = 153	N = 882	
Mechanical valve	65.5	51.6	67.9	<0.001
	(62.5 – 68.4)	(43.4 – 59.8)	(64.7 – 71.0)	
Bioprosthetic valve	24.5	26.8	24.1	0.482
	(21.9 – 27.3)	(20.0 – 34.5)	(21.4 – 27.1)	
Valve Repair	10	21.6	7.9	<0.001
	(6.4 – 14.8)	(15.3 – 28.9)	(6.2 – 9.9)	
Aortic		N = 62	N = 628	
Mechanical valve	53.6	64.5	52.5	0.071
	(49.8 – 57. 4)	(51.3 – 76.3)	(48.6 – 56.5)	
Bioprosthetic valve	44.2	32.3	45.4	0.047
	(40.5 – 48.0)	(20.9 – 45.3)	(41.4 – 49.4)	
Valve Repair	2.2	3.2	2.1	0.552
	(1.22 – 3.56)	(0.4 – 11.2)	(1.1 – 3.5)	
Tricuspid		N = 31	N = 188	
Mechanical valve	10	3.2	11.2	0.173
	(8.2 – 11.9)	(0.1 – 16.7)	(7.0 – 16.6)	
Bioprosthetic valve	3.7	6.5	3.2	0.370
	(1.6 – 7.1)	(0.8 – 21.4)	(1.2 – 6.8)	
Valve Repair	86.3	90.3	85.6	0.482
	(81.0 – 90.6)	(74.2 – 98.0)	(79.8 – 90.3)	

Multivariate logistic regression modeling was undertaken to identify independent predictors of mechanical versus bioprosthetic valve replacement and valve replacement versus repair. In patients having an RHD-related valve replacement, mechanical valves, compared with only using bioprosthetic valves, were more likely to be used in those with associated AF (OR 2.69, 95% CI 1.64 – 4.43), when more than one valve required surgery (OR 1.61 for each additional valve, 95% CI 1.03 – 2.49) and if there was a history of previous PBV (OR 3.12, 95% CI 1.87 – 5.21) or other valve surgery (OR 3.12, 95% CI 1.87 – 5.21). Mechanical valves were less likely to be used in those with diabetes (OR 0.51, 95% CI 0.29 – 0.89) or chronic kidney disease (OR 0.50, 95% CI 0.30 – 0.83). Whilst the median age of those receiving mechanical valves was significantly lower (57.1 years, IQR (50.0 – 67.1)) than for those receiving bioprosthetic valves (65.8 years, (61.2 – 77.0), p = <0.001), this was not significant after adjusting for these other covariates. Indigenous status and remoteness of residence were not significant predictors of valve type choice.

In multivariate modelling, patients having isolated valve repair, compared with any valve replacement, were more likely to be Aboriginal and Torres Strait Islander (OR 5.50, 95% CI 3.24 – 9.35), to have fewer valves requiring surgery (OR 0.10 for each additional valve, 95% CI 0.04 – 0.28) and were less likely to have hypertension (OR 0.53, 95% CI 0.32 – 0.89) or a history of smoking (OR 0.59, 95% CI 0.37 – 0.96). Whilst patients having isolated valve repair, compared with any valve replacement were more likely to be younger, reside in a remote area and less likely to have associated AF or concomitant CABG, these were not significant predictors after adjusting for other significant covariates.

**Figure 3:**
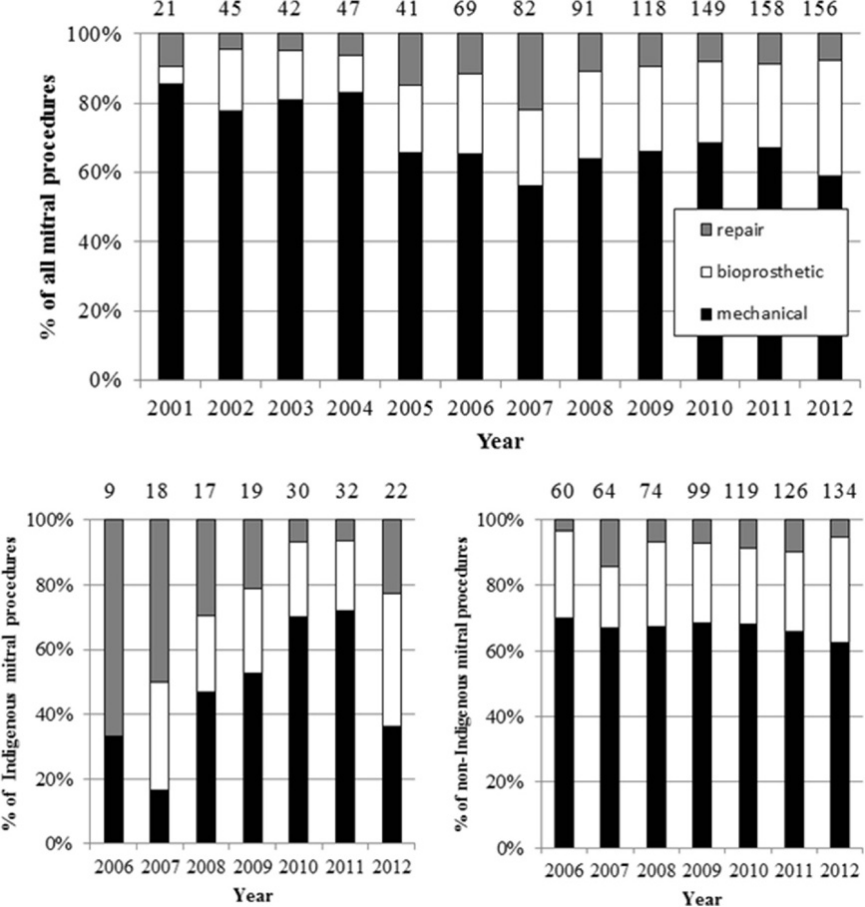
**Changes in RHD mitral valve surgery over time, total and stratified by Indigenous status.** (Numbers at the top of each column refer to the total number of procedures for that year).

**Figure 4:**
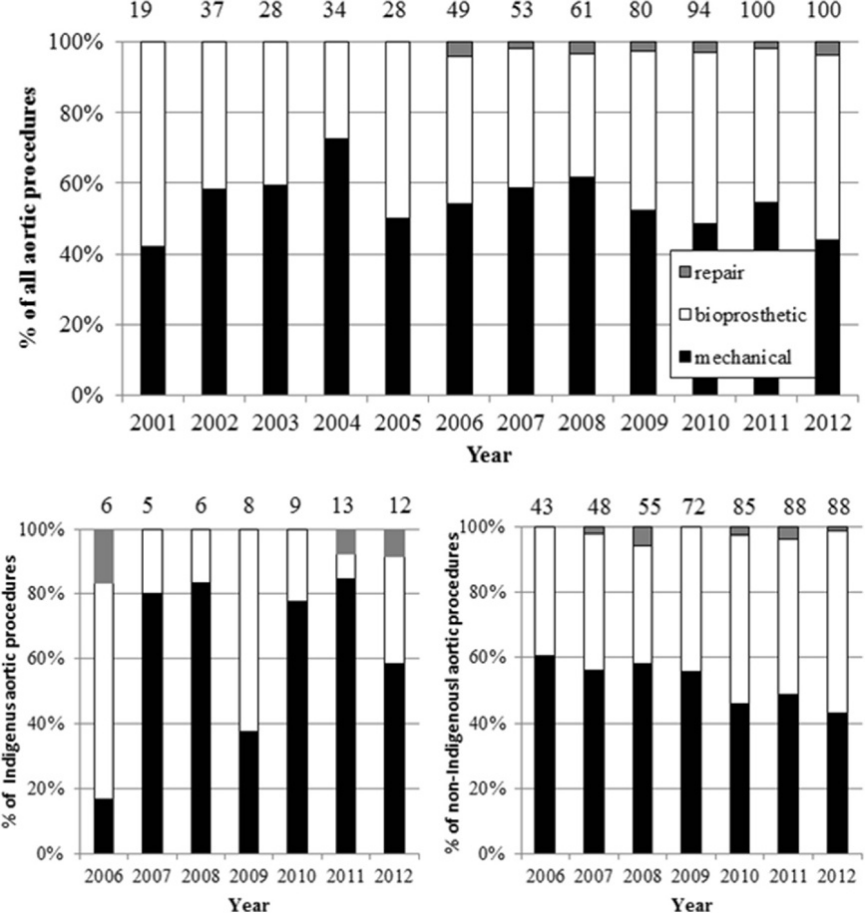
**Changes in RHD aortic valve surgery over time, total and stratified by Indigenous status.** (Numbers in each column refer to the total valve surgeries for that year).

Temporal trends in the surgical management of RHD-related mitral and aortic valve disease are outlined in Figures 3 and 4. Overall there was no significant change in aortic valve surgery type over this time. Mitral valve procedures demonstrated a significant increase in bioprosthetic valve replacements (1.8% increase as a proportion of all mitral valve procedures/year, 95% CI 1.0 – 2.6) and a corresponding fall in mechanical valve replacements (1.8% decrease/year, 95% CI 1.0 – 2.6). Whilst mitral valve repairs decreased (1.0% decrease/year, 95% CI −0.5 – 1.7) this was not statistically significant. Given major centres undertaking valve surgery for Aboriginal and Torres Strait Islander peoples only began submitting data from 2006, analysis of temporal trends in the choice of valve surgery stratified by Indigenous status was restricted to 2006 – 2012. Analysis of mitral procedures over time revealed mitral valve repairs declined (Spearman rank r = −0.786, p = 0.036) in Aboriginal and Torres Strait Islander patients from 2006–2012. Aortic valve procedures in non-Indigenous Australian patients over the same time demonstrated an increase in the use of bioprosthetic valves (Spearman rank r = 0.857, p = 0.014) and a decrease in mechanical valves (Spearman rank r = −0.929, p = 0.003). The surgical management of mitral valve disease in non-Indigenous Australians and aortic valve disease in Aboriginal and Torres Strait Islander peoples did not alter significantly over this time.

The nature of the underlying RHD-related mitral and aortic valve lesions stratified by Indigenous status are outlined in Table 5. Aboriginal and Tosses Strait Islander people were, compared with non-Indigenous Australians, more likely to have only mitral stenosis and regurgitation as well as mixed mitral disease and, whilst more likely to have isolated aortic regurgitation, were less likely to have aortic stenosis only or mixed aortic disease.

**Table 5:** **RHD Mitral and aortic valve lesions types stratified by Indigenous status**

% (95% CI)	Total RHD	Aboriginal and/or Torres Strait Islander	Non-Indigenous Australian	P value
	N = 1384	N = 174	N = 1210	
Mitral		n = 152	n = 882	
Stenosis only	5.3	12.1	4.3	<0.001
(95% CI)	(4.2 – 6.6)	(7.6 – 17.9)	(3.2 – 5.6)	
Regurgitation only	21	31.0	19.5	<0.001
(95% CI)	(18.8 – 23.2)	(24.3 – 38.5)	(17.3 – 21.9)	
Combined regurgitation and stenosis	28.8	36.2	27.8	0.022
(95% CI)	(26.5 – 31.3)	(29.1 – 43.8)	(25.3 – 30.4)	
Aortic valve		n = 62	n = 628	
Stenosis only	4.9	1.1	5.5	0.014
(95% CI)	(3.8 – 6.2)	(0.1 – 4.1)	(4.2 – 6.9)	
Regurgitation only	12.1	21.8	10.7	<0.001
(95% CI)	(10.4 – 13.9)	(15.9 – 28.7)	(9.0 – 12.5)	
Combined regurgitation and stenosis	17.1	12.1	17.8	0.052
(95% CI)	(15.1 – 19.1)	(7.6 – 17.9)	(15.7 – 20.0)	

The utilisation of mitral or aortic valve repair as compared with valve replacement stratified by the underlying valve lesion is presented in Table 6. Mitral valve repair was more likely to be undertaken in isolated regurgitation and mixed disease and aortic valve repair in those with mixed disease.

**Table 6:** **RHD Mitral and aortic valve lesions types stratified by isolated repair or replacement**

% all valve procedures (95% CI)	Isolated valve repair N = 74	Any valve replacement N = 1297	P value repair versus replacements
Mitral			
Stenosis only	6.8	5.2	0.573
(95% CI)	(2.2 – 15.1)	(4.1 – 6.6)	
Regurgitation only	54.1	19.0	<0.001
(95% CI)	(42.165.7)	(16.9 – 21.2)	
Combined regurgitation and stenosis	14.9	29.8	0.006
(95% CI)	(7.7 – 25.0)	(27.3 – 32.3)	
Aortic valve			
Stenosis only	1.4	5.2	0.142
(95% CI)	(0.03 – 7.3)	(04.0 – 6.5)	
Regurgitation only	9.5	12.3	0.462
(95% CI)	(3.9 – 18.5)	(10.6 – 14.3)	
Combined regurgitation and stenosis	5.4	17.8	0.006
(95% CI)	(1.5 – 13.3)	(15.7 – 20.0)	

## Discussion

This study is the first to provide a detailed description of Australian RHD valve surgery. It analysed 17227 patients in Australia who had surgical valve procedures performed between 2001 and 2012 including 1384 RHD valve procedures. The high burden of RHD among Aboriginal and Torres Strait Islander people was reflected in the relatively high percentage of Indigenous Australians requiring RHD surgery (12.6%) compared with their representation in the overall Australian population (2.5%) [19]. Advanced RHD affects people at a younger age compared with non-RHD-related valve disease [20, 21]. Australians in general and Aboriginal Australian and/or Torres Strait Islander in particular required RHD valve surgery at a younger age, a finding reflected in several earlier studies [9, 22].

The finding that RHD surgery patients were both younger and more likely to be female has implications for treatment choice, particularly given the potential hazards of anticoagulation associated with future pregnancies [23]. It is also of relevance for other younger people who often participate in activities associated with an increased risk of trauma (e.g. contact sports). AF was more common in those having RHD-related compared to non-RHD-related valve surgery. Undertaking RHD mitral valve surgery prior to the onset of AF would appear to provide greater therapeutic choice as both bioprosthetic valve replacement and valve repair do not typically require ongoing anticoagulation in the presence of sinus rhythm and no embolic history. This can be particularly useful when managing younger and female patients for the reasons outlined above and for Indigenous Australian patients who are more likely to reside in remote communities where anticoagulation monitoring and ongoing specialist review can be difficult. Nonetheless the associated increased risk of surgical re-operation in valve repair and bioprosthetic valve replacement must also be considered in the decision-making process.

There was an independent association between RHD valve surgery and previous PBV for mitral stenosis. This is not surprising given PBV can often provide temporary relief of mitral stenosis with restenosis being reported in a number of studies, ranging from 40% of patients at six years [24], 34% at 10 years [25] and 21% at 15 years [26, 27]. Despite this risk of restenosis, PBV can provide a non-invasive approach to mitral stenosis management that does not necessarily require ongoing anticoagulation and which has excellent overall survival rates ranging from 96.5% at three years [24] to 99.2% at 16 years [26].

Aboriginal Australian and/or Torres Strait Islander people were less likely to have concomitant CABG when having RHD-related valve surgery. This is surprising given Indigenous Australians are hospitalised 1.9 times more than non-Indigenous Australians for coronary heart disease [28]. Nonetheless this may, at least in part, be explained by the younger age of Indigenous Australian RHD patients who had a median age nearly 30 years less than that of non-Indigenous patients.

We also found Indigenous Australian RHD patients were less likely to have associated kidney disease. This is also perhaps unexpected given the well-documented epidemic of kidney disease in Aboriginal and Torres Strait Islander people [29]. Nonetheless this finding did not persist in multivariate analysis, suggesting the older age of non-Indigenous Australian RHD patients had a greater effect on chronic kidney disease risk compared with younger Aboriginal and Torres Strait Islander patients. This finding is not universal and Indigenous Australian cardiac surgical patients have reported to have an increased burden of kidney disease pre-operatively [7, 8]. These reports are likely to have represented Aboriginal and Torres Strait Islander populations which may have been at greater risk of chronic kidney disease due to the high proportion of patients residing in remote centres where the risk of kidney disease has also been shown to be greater [29, 30]. Such disparity between these earlier single centre studies and our larger multicenter review reinforces the benefits of national data collection systems such as that used here.

The valves involved in RHD valve surgery were in line with earlier studies, most commonly the mitral and aortic valves, less commonly the tricuspid and rarely the pulmonary valve. Isolated RHD-related tricuspid valve disease is relatively uncommon [31] and represented only 1.2% of Australian patients having RHD valve surgery. Thirty percent of patients having RHD-related surgery required management of multiple valves, highlighting the increased complexity of surgery in RHD-related valve disease.

The choice of valve procedure is likely to be informed by a combination of patient, health practitioner choice, demographic and disease factors. A mechanical valve has long term durability providing therapeutic anticoagulation can be achieved compared to a bioprosthetic valve which is likely to degenerate over time [1, 23, 32, 33]. Mechanical valves may therefore be preferred in younger patients so as to avoid later re-operation. Nonetheless this must be balanced against the inconvenience and risk of anticoagulation in a younger patient who may wish to become pregnant or to participate in recreational or employment activities that entail a greater risk of trauma. The balancing of these factors means there is no universally correct approach to treatment choice in the individual patient. Our data would suggest that mechanical valves are preferred in younger patients irrespective of whether they are Indigenous or not. This is particularly the case when there is co-existent AF (and therefore an additional indication for anticoagulation) and the patient has represented following earlier PBV or past valve surgery. Whilst such an approach can be argued as potentially reasonable for patients living in remote Australia, it would suggest that decisions regarding the use of mechanical valves, particularly in younger Aboriginal and Torres Strait Islander people should be undertaken cautiously and in association with the patient, their family, community and local health care providers. The difficulty of maintaining long-term anticoagulation, particularly in a remote setting, should not be underestimated. In a review of RHD patients prescribed warfarin, 37% had inadequate monitoring and 65% of INR results were outside the recommended range [34]. Our findings support an increasing preference for bioprosthetic over mechanical valve replacement for mitral valve disease and may reflect a greater appreciation of the factors outlined above. Variability in local management practices including the timing of surgical referral and surgical centre practices and expertise are also likely to influence the timing and type of surgery performed. Earlier referral to a surgical centre with a specific interest in valve repair is thus likely to increase the possibility of repair.

Mitral valve repairs as a proportion of all mitral valve procedures decreased (1.0% decrease/year, 95% CI −0.5 - 1.7) but this was not statistically significant. Mitral valve repair compared to replacement has previously been associated with higher survival rates [35–37] in young RHD patients, with Remenyi et al. [35] reporting actuarial survival at 10 and 14 years for patients with mitral replacement of 79% and 44%, compared to 90% and 90% for those who underwent mitral repair. Similarly Wang et al. [37] in a systematic review of mitral valve repair and replacement found a survival benefit associated with mitral repair over replacement.

Not all valves, however, are suitable for repair [38] and repaired valves have an increased risk of early reoperation [38, 39]. A key factor in increasing the chance of successful mitral valve repair is likely to be earlier referral prior to the onset of valvular fibrosis and calcification which may reduce the chance of successful repair [40] and concentrating RHD surgical management in centres with greater experience in this area.

Whilst the proportion of mitral valve procedures that were repairs rather than replacements had not significantly altered over time it was noted that for all mitral valve lesions, not just mitral regurgitation, that mitral valve repair was more likely in Indigenous patients. This is likely to reflect an understanding of the difficulties associated with anticoagulation. Non-Indigenous patients are more likely to reside in metropolitan Australia and to be more likely to be able to achieve safe anticoagulant use and monitoring. In such a setting mechanical valve replacement with attendant long-term anticoagulation is likely to be preferable. The utilization of mitral valve repair in mitral stenosis and mixed mitral valve disease demonstrates the diversity of valvular lesions that are encountered and dealt with by surgeons when dealing with RHD and the broad scope of expertise required.

### Limitations of the study

The main limitation of this study is that it is restricted to Australian surgical practice and does not reflect management in other countries. Nonetheless overall this sample is likely to provide an accurate representation of surgical management of RHD in Australia. Whilst the ANZSCTS database receives data from 19 Australian public hospitals there are six public hospitals in Australia which perform cardiac surgery but do not provide data. It is unlikely the inclusion of these centres would have significantly altered our findings. Of particular note is the inclusion of data from the major Australian centres performing RHD-related valve surgery in Aboriginal Australian and Torres Strait Islander patients. The multiple data collection sites may have led to variable data coding. This was however minimised by each site employing its own data manager who was supported with training and standard data definitions, the use of standardised data entry systems and centralised auditing of site-specific data.

## Conclusions

This study is one of the largest reviews of patients undergoing RHD valve surgery. Mitral and aortic valve disease remains the focus of most surgery but tricuspid valve procedures are not uncommon. A range of factors have been identified which are associated with particular surgical procedures. Whilst many of these reflect the underlying nature of disease, the role of AF in predicting treatment choice would suggest that earlier surgery, prior to the onset of AF, and more aggressive management of AF if it does occur, may allow a broader choice of intervention and, correspondingly, less requirement for life-long anticoagulation. Whilst mechanical valves were more likely to be used in younger patients, this needs to be balanced against fertility, lifestyle planning and the safety of anticoagulant use particularly in younger, remote and Aboriginal and Torres Strait Islander patients. The greater use of bioprosthetic valves, valve repair and PBV, whilst having a greater risk of reoperation, may be more suitable in such patients. Earlier referral and surgical management of such patients to centres with expertise in managing RHD valve disease is likely to provide greater opportunity for valve repair and PBV.

## Abbreviations

RHD: Rheumatic heart disease
AF: Atrial fibrillation
PBV: Percutaneous balloon valvuloplasty
ANZSCTS: Australia and New Zealand Society of Cardiac and Thoracic Surgeons
CABG: Coronary artery bypass grafting
eGFR: Estimated glomerular filtration rate
MDRD: Modification of Diet in Renal Disease
NYHA: New York Heart Association
LVEF: Left ventricular ejection fraction
CI: Confidence interval
SD: Standard deviation
IQR: Interquartile range
OR: Odds ratio.
